# Comparative Studies of g-C_3_N_4_ and C_3_N_3_S_3_ Organic Semiconductors—Synthesis, Properties, and Application in the Catalytic Oxygen Reduction

**DOI:** 10.3390/molecules28062469

**Published:** 2023-03-08

**Authors:** Ewelina Wierzyńska, Marcin Pisarek, Tomasz Łęcki, Magdalena Skompska

**Affiliations:** 1 Faculty of Chemistry, University of Warsaw, Pasteura 1, 02-093 Warsaw, Poland; 2 Institute of Physical Chemistry, Polish Academy of Sciences, Kasprzaka 44/52, 01-224 Warsaw, Poland

**Keywords:** g-C_3_N_4_, C_3_N_3_S_3_, band diagrams, oxygen reduction

## Abstract

Exfoliated g-C_3_N_4_ is a well-known semiconductor utilized in heterogenous photocatalysis and water splitting. An improvement in light harvesting and separation of photogenerated charge carriers may be obtained by polymer doping with sulfur. In this work, we incorporate sulfur into the polymer chain by chemical polymerization of trithiocyanuric acid (C_3_N_3_S_3_H_3_) to obtain C_3_N_3_S_3_. The XRD measurements and TEM images indicated that C_3_N_3_S_3_, in contrast to g-C_3_N_4_, does not exist in the form of a graphitic structure and is not exfoliated into thin lamellas. However, both polymers have similar optical properties and positions of the conduction and valence bands. The comparative studies of electrochemical oxygen reduction and hydrogen evolution indicated that the overpotentials for the two processes were smaller for C_3_N_3_S_3_ than for g-C_3_N_4_. The RDE experiments in the oxygen-saturated solutions of 0.1 M NaOH have shown that O_2_ is electrochemically reduced via the serial pathway with two electrons involved in the first step. The spectroscopic experiments using NBT demonstrated that both polymers reveal high activity in the photocatalytic reduction of oxygen to superoxide anion radical by the photogenerated electrons.

## 1. Introduction

Graphitic carbon nitride (g-C_3_N_4_) is one of the most popular organic semiconductors utilized in heterogeneous photocatalysis [1,2,3]. It may be synthesized by thermal condensation of cyanamide, dicyandiamide, melamine, thiourea, and urea ([4] and the references therein). The band gap of g-C_3_N_4_, 2.7 eV, is small enough to absorb the photons from the visible light but sufficiently large to fulfill the thermodynamic requirements for water splitting. Namely, the bottom of the conduction band is located above the redox potential of the H^+^/H_2_ couple, while the edge of the valence band is more positive than the oxidation potential of water to O_2_ [5]. Moreover, the microstructure of g-C_3_N_4_ with a large number of termination atoms and defects is beneficial for anchoring the active sites. Therefore, the bulk g-C_3_N_4_ is exfoliated in aqueous (acidic or basic solutions) or organic solvents to obtain ultrathin or monolayer nanosheets of more abundant surface active sites [6,7] and very high specific surface area.

Using g-C_3_N_4_ as a metal-free photocatalyst for water splitting to H_2_ under visible light, with triethanolamine as the hole scavenger, has been first reported by Wang et al. [8]. However, an efficient and stable hydrogen evolution was possible after modification of g-C_3_N_4_ with a small amount (3 wt%) of Pt co-catalyst. It was also found that some improvement in the photocatalytic properties of g-C_3_N_4_ could be achieved by molecular doping. For example, the band gap energy of sulfur-doped g-C_3_N_4_ (S-g-C_3_N_4_) is reduced to 2.63 eV [9], which allows better utilization of the solar spectrum. The theoretical calculations predicted equal band gaps of g-C_3_N_4_ and S-g-C_3_N_4_, but the S-doped polymer has the impurity level located in the band gap. This ensures easy excitation of electrons from the valence band to the impurity level. Launching sulfur into the g-C_3_N_4_ structure also improves the charge carrier separation and prevents recombination. The electrons from sulfur-doped g-C_3_N_4_ have been utilized to reduce CO_2_ to obtain CH_3_OH, while the holes from the valence band were involved in water oxidation [9]. The hybrid system of TiO_2_/S-g-C_3_N_4_ was used in the photocatalytic degradation of Congo Red [10]. The significant enhancement of photocatalytic activity was ascribed to the S-scheme mechanism and well-distributed 1D nanostructure of doped polymer.

Another possibility of sufur incorporation into the polymer chain is the synthesis from the monomer containing sulfur in the molecular structure. Therefore, in this work, we synthesized C_3_N_3_S_3_ by chemical polymerization of trithiocyanuric acid (C_3_N_3_S_3_H_3_) and compared the properties of the obtained organic semiconductor with those of g-C_3_N_4_ formed by thermal condensation of urea. It has been shown that both polymers reveal ambipolar properties, i.e., behave as n-type or p-type semiconductors, depending on the polarization range. The band diagrams of both semiconductors were constructed taking into account the position of the Fermi level determined from photocurrent onset potential, and the valence band position was verified by VB X-ray photoelectron spectra. The formation of superoxide anion radicals by the electrons photogenerated in the conduction bands of both semiconductors was confirmed by the Nitro Blue Tetrazolium chloride (NBT) experiments. Finally, both polymers were used in the reactions of oxygen reduction and electrocatalytic hydrogen generation.

## 2. Results

### 2.1. Characterization of C_3_N_3_S_3_

**Figure 1:**
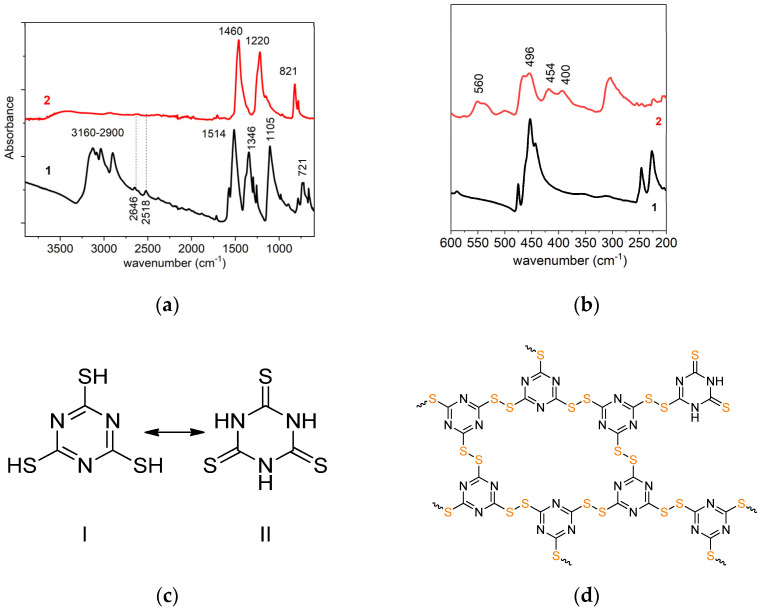
FTIR spectra of the monomer C_3_N_3_S_3_H_3_ (lines 1) and the polymer C_3_N_3_S_3_ (lines 2) in the wavenumber range 3800 cm^−1^–600 cm^−1^ (**a**) and 600 cm^−1^–350 cm^−1^ (**b**); the chemical structures of the monomer in the tautomeric form of thiol (I) and thione (II) (**c**), and probable structure of the polymer C_3_N_3_S_3_ (**d**).

The successful synthesis of C_3_N_3_S_3_ polymer by chemical oxidation of C_3_N_3_S_3_H_3_ with I_2_ has been confirmed by FTIR spectra presented in Figure 1.

In the spectrum of the monomer (line 1), one can observe several sharp peaks in the range 3160–2900 cm^−^^1^, characteristic for N-H stretching vibrations in triazine groups in the monomer [11] (Figure 1c), which are not visible in the spectrum of the polymer (line 2). The peaks at 1514, 1346, 1105, and 721 cm^−^^1^ in the monomer spectrum are ascribed to the stretching vibrations of non-aromatic heterocycle thione (tautomeric structure II in Figure 1c). Specifically, the bands at 1514 and 1346 cm^−^^1^ may be ascribed to C=N and C-N stretching vibrations [12] in the triazine ring, while that at 1105 cm^−^^1^ is assigned to C=S stretching vibrations [13]. In the polymer, the peaks at 1514, 1346, and 720 cm^−^^1^ are shifted to 1460, 1220, and 821 cm^−^^1^, respectively, while the trace of the C=S peak is observed in the band shoulder at about 1140 cm^−^^1^. It likely origins from the thione groups in the external, terminal units of the polymer network. The formation of the polymer is supported by the presence of new peaks in the range 560–400 cm^−^^1^ (Figure 1b), ascribed to disulfide S-S linkages, and the disappearance of two weak peaks at 2518 and 2646 cm^−^^1^ corresponding to S-H stretching. The peak at about 450 cm^−^^1^ observed both in the spectrum of the monomer and polymer originates from NCN bending vibration [14].

**Figure 2:**
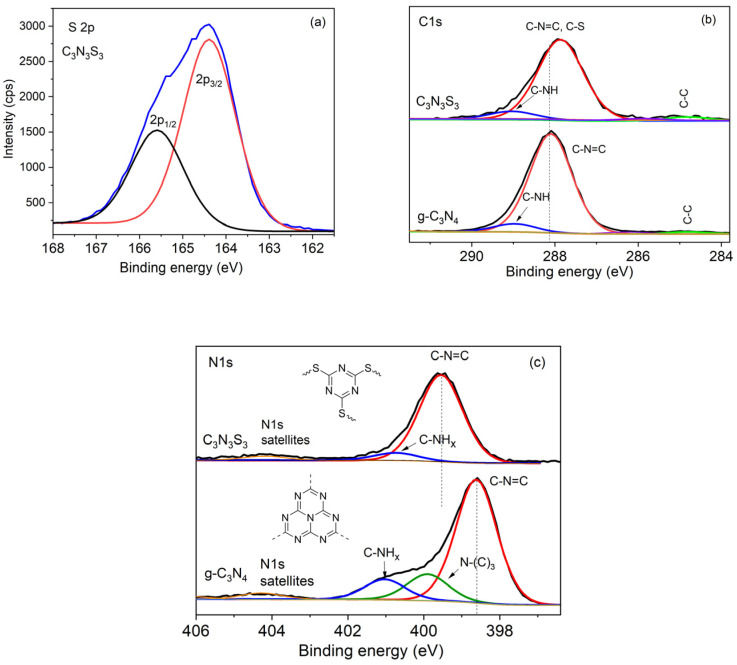
High-resolution XPS spectrum of S 2p of C_3_N_3_S_3_ (**a**), and comparison of HR-XPS spectra of C 1s (**b**), N 1s (**c**) of C_3_N_3_S_3_ and g-C_3_N_4_.

The successful polymerization of thiocyanuric acid to C_3_N_3_S_3_ has also been confirmed by XPS measurements. The main peaks in the survey spectrum presented in Figure S1 originate from C 1s, N 1s, and S 2p. Two additional peaks of very small intensities were detected at the binding energies 621.2 eV and 632.5 eV (Figure S2a), corresponding to I 3d_5/2_ and I 3d_3/2_, originate from the traces of I_2_ oxidant (about 0.2 at.%) used for polymerization. The formation of S-S bonds in the polymer is manifested in the high-resolution XPS spectrum (Figure 2a) by two peaks at the binding energies 164.7 eV and 165.9 eV, ascribed to S 2p_3/2_ and S 2p_1/2_, respectively [15,16].

In the deconvoluted spectrum of C_3_N_3_S_3_, the main C 1s peak at the binding energy 287.9 eV, corresponding to N-C=N and C-S bonds (Figure 2b), is shifted by about 0.2 eV towards lower energies with respect to the C 1s peak ascribed to sp^2^-bonded carbon in N-C=N, in the spectrum of g-C_3_N_4_, due to the presence of sulfur in the chemical environment of carbon. There is also a small component of C 1s peak in the spectra of both polymers at about 289 eV, which may be ascribed to sp^2^ C bonded to the NH group (sp^2^ C-NH) [17]. On the other hand, the signal at this binding energy may also originate from oxygen-carbon functional groups (C=O and C-O) due to the presence of surface carboxylic groups. However, the O 1s peaks of very small intensity were detected only in the spectrum of C_3_N_3_S_3_ (see the survey and high-resolution spectra in Figures S1a and S2b) but not for g-C_3_N_4_ (Figure S1b).

The significant differences in the spectra of the two polymers are observed for the peak N 1s (Figure 2c). The peak N 1s for C_3_N_3_S_3_ is narrow because it has only two components: the peak at 399.6 eV corresponding to sp^2^-hybridized nitrogen in C-N=C, and a small peak at about 400.7 eV corresponding to C-NH_x_, due to the presence of the external nitrogen atoms in the polymer network (Figure 1d). In contrast, the N 1s peak in the spectrum of g-C_3_N_4_, with a broad shoulder on the higher energy side, can be deconvoluted in three components. The main peak located at about 398.6 eV corresponds to sp^2^-hybridized nitrogen (C-N=C), while the component at about 400 eV is ascribed to sp^3^ tertiary/bridging N-(C)_3_ nitrogens [18] (not present in the case of C_3_N_3_S_3_). A weak peak at the binding energy ~401.0 eV is attributed to the nitrogen in terminal amino groups, C-NH_x_. In the spectra of both polymers, there are also very small peaks at the binding energy of about 404.0 and 407.0 eV, which correspond to shake-up satellite peaks of N 1s. These data are in good agreement with the XPS observations and DFT calculations of Zhang and co-authors for different g-C_3_N_4_ models [19]. Such satellite signals can also be observed for carbon C 1s.

The atomic ratios obtained from XPS data are C:N = 1:1.27 for g-C_3_N_4_ (being very close to the theoretical ratio 1:1.3) and C:N:S = 1:0.84:1.1 for C_3_N_3_S_3_ (see details in Tables S1 and S2 in SM).

**Figure 3:**
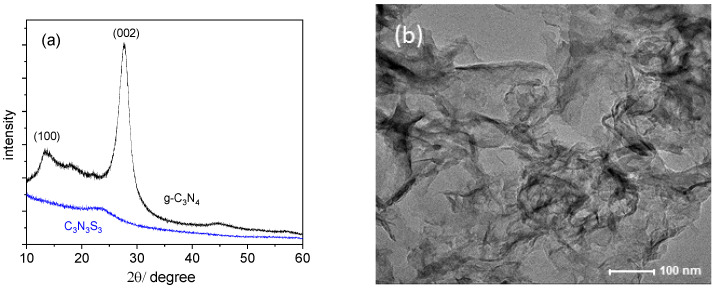

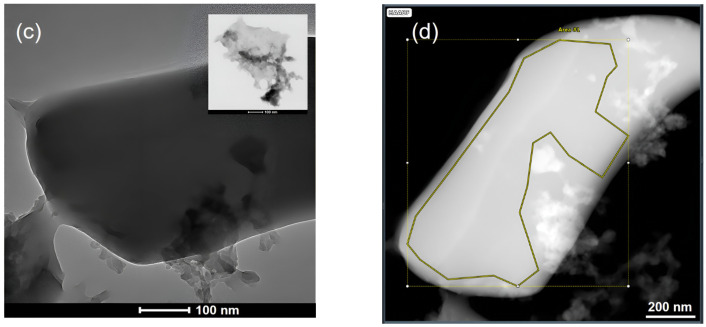
The XRD patterns of C_3_N_3_S_3_ and g-C_3_N_4_ (**a**); TEM images of g-C_3_N_4_ (**b**) and C_3_N_3_S_3_ (**c**) after sonication in DMSO, and HAADF image of C_3_N_3_S_3_ with the indicated area of elemental analysis (**d**).

The XRD patterns presented in Figure 3a indicate the significant differences in the crystallographic structures of C_3_N_3_S_3_ and g-C_3_N_4_.

The XRD pattern of g-C_3_N_4_ is consistent with the reference diffractogram in the JCPDS (no. 87-1526) [20]. A high signal at 2θ of about 27° is indexed as (002) peak originating from the interplanar diffraction, confirming the graphitic-like structure of this polymer [8]. It corresponds to the interlayer distance of d = 3.36 Å, which is comparable to the packing in the crystalline graphite. The second peak at about 13° of markedly lower intensity, indexed as (100), is ascribed to in-planar repeated triazine units [21]. In contrast, the diffractogram of C_3_S_3_N_3_ with only one broad peak at about 23°, similar to that reported in the literature [16], is typical for amorphous material. Different morphology of C_3_S_3_N_3_ and g-C_3_N_4_ results in different behavior of the two polymers during sonication in DMSO used as the solvent. As visible in TEM images of the polymers sonicated for 10 h, the g-C_3_N_4_ underwent exfoliation into lamellas (Figure 3b), while C_3_S_3_N_3_ still existed in the form of thicker flakes of micrometer length and smooth surface. However, in the latter case, one can observe the thin flakes or aggregated particles attached to the large and flat elements (see inset in Figure 3c).

The atomic ratio C:N:S obtained for the area marked in Figure 3d is about 1:0.8:1.1, which is practically the same as the ratio obtained from XPS measurements (1:0.84:1:1), and in both cases, the amount of N in C_3_N_3_S_3_ is a little lower than the expected one, while the relative S content is a little too high. However, EDS elemental maps have shown that all elements are uniformly distributed over the whole sample (Figure S3).

### 2.2. Determination of the Band Diagrams of g-C_3_N_4_ and C_3_N_3_S_3_

**Figure 4:**
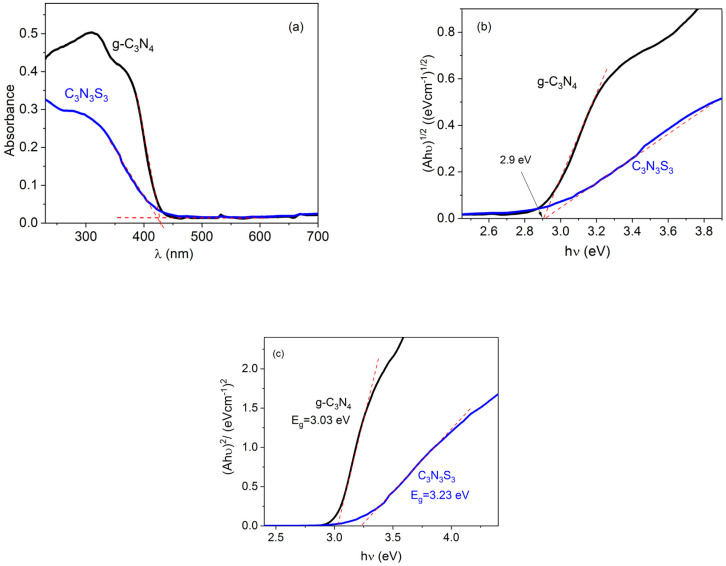
UV-Vis absorption spectra (**a**) and Tauc plots for C_3_N_3_S_3_ and g-C_3_N_4_ for indirect (**b**) and direct (**c**) band transitions.

According to the UV-Vis absorption spectra presented in Figure 4a, the optical absorption edges of g-C_3_N_4_ and C_3_N_3_S_3_ are at the same wavelength, 430 nm. This wavelength corresponds to the band gap energy 2.88 eV calculated from the equation *E_g_* (eV) = 1240/λ(nm).

The band gap of both materials was also determined from the Tauc plot:

(1) $${\left(\alpha h\nu \right)}^{1/n}=A\left(h\nu -E_{g}\right)$$

where *α* is an absorption coefficient, *h* is the Planck constant, *ν* is the photon’s frequency, and *A* is a constant. The value of factor n is equal to 1/2 for direct band transition and 2 for indirect transition [22]. The g-C_3_N_4_ is reported in the literature as an indirect semiconductor [23,24,25], while no data on the type of transition is available for C_3_N_3_S_3_. Therefore, the Tauc plots were done for *n* = 2 (Figure 4b) as well as for *n* = 1/2 (Figure 4c). Since the value of *E_g_* = 2.9 eV, obtained for both polymers from the plot ${\left(\alpha h\nu \right)}^{1/2}\;vs\;h\nu$, is close to band gap energy determined directly from UV-Vis spectrum (Figure 4a), one can conclude that the transition in the C_3_N_3_S_3_ is also indirect. The band gap energy obtained for g-C_3_N_4_ is a little higher than the values reported in the literature (2.6–2.85 eV) [6,8,26], which may be explained by lower temperature applied during the precursor condensation (500 °C) than most often applied in the literature (550–600 °C). It has been reported that the decrease of the band gap energy with the increase of the processing temperature is related to a gradual increase in the polymerization degree [27]. On the other hand, the increase of the synthesis temperature above 550 °C may lead to some distortion of the (100) crystal plane and a decrease in the photocatalytic ability of g-C_3_N_4_ [20].

The experimental data on C_3_N_3_S_3_ are very scarce, but according to DFT calculations, the band gap of this polymer may vary from 3.77 eV to 1.9, depending on the polymerization degree [16].

The edge of the conduction band of g-C_3_N_4_ is often estimated in the literature from the Butler and Ginley relationship [28]:

(2) ECB[vs SHE]=1e(χ−12Eg−Ee) [eV]

where *E^e^* is the energy of free electrons with respect to SHE (0 V vs. *SHE* corresponds to 4.5 eV), *E_g_* is the band energy, and *χ* is the semiconductor electronegativity, which is defined as the geometric mean of the electronegativities of the constituent neutral atoms *χ*(M). However, according to Praus [29], this method is not relevant for the determination of the band edges of g-C_3_N_4_ since this material is not characterized by a single value of electronegativity due to possible structural defects, layer distortions, and the presence of natural impurities, such as oxygen, etc. It has been shown that the *χ* obtained for g-C_3_N_4_ (6.91 eV) leads to the band edges E_CB_ and E_VB_ far from those obtained from experimental values [29]. Recently, we have reported the same problem with the application of the Butler and Ginley approach in the determination of correct potentials of the band edges of BiVO_4_ [30].

Experimentally, the conduction band of the semiconductors is most often determined from the flat band potential (E_fb_) obtained from the Mott–Schottky plot [31]. However, the main assumption made in the derivation of the Mott–Schottky equation, such as a perfectly planar semiconductor surface or a homogeneous distribution of electronic defects, is not fulfilled in the case of lamellar g-C_3_N_4_. Alternatively, the value of E_fb_ may be determined from the onset potential of photocurrent by analysis of the voltammograms in the dark and under illumination [32,33], and this method gave very reliable results for Fe_2_O_3_ [33] and BiVO_4_ [30].

**Figure 5:**
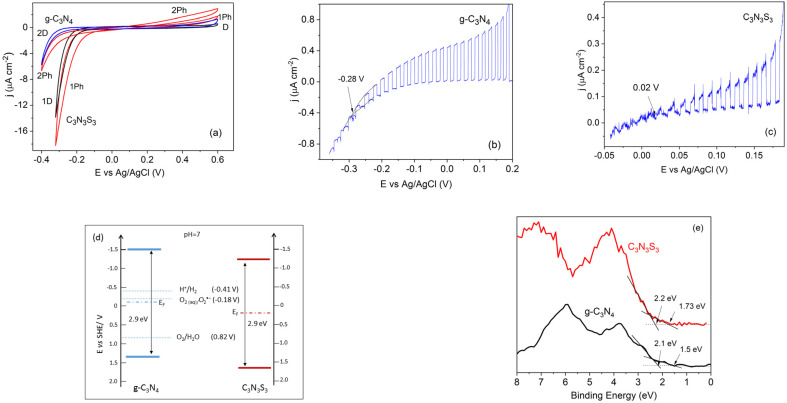
Cyclic voltammograms for C_3_N_3_S_3_ (lines 1) and g-C_3_N_4_ (lines 2) in the dark (D) and under illumination (Ph) in the solution of 0.1 M Na_2_SO_4_ (**a**); linear sweep voltammograms (LSVs) for g-C_3_N_4_ (**b**) and C_3_N_3_S_3_ (**c**) at the sweep rate of 1 mV s^−^^1^ under chopped illumination in a deaerated solution of 0.1 M Na_2_SO_4_; the band energy diagrams of g-C_3_N_4_ and C_3_N_3_S_3_ constructed on the base of electrochemical data with the redox potentials of the solution species at pH 7 (**d**), and VB XP spectra of C_3_N_3_S_3_ and g-C_3_N_4_ (**e**).

The cyclic voltammograms of FTO/g-C_3_N_4_ and FTO/C_3_N_3_S_3_ electrodes obtained in the solution of 0.1 M Na_2_SO_4_, presented in Figure 5a, indicate that both polymers exhibit an ambipolar behavior, i.e., as a p-type semiconductor with negative photocurrents under cathodic bias, and n-type with positive photocurrents in the range of anodic potentials. This type of behavior is typical for organic semiconductors, as well as two-dimensional layered materials, such as metal dichalcogenides [34]. It has also been reported for g-C_3_N_4_ as amphoteric behavior [35,36]. As visible in Figure 5a, the anodic photocurrent densities for both polymers studied were markedly lower than the reduction photocurrents. This may be explained by considering the electrode–solution interface reactions. In the range of positive potentials, the photogenerated holes may be involved in water oxidation. However, this reaction is rather complex because it requires the transfer of four electrons to oxidize two H_2_O molecules with the removal of four protons to form the O=O bond [37]. In contrast, the cathodic current for both polymers was high, also without illumination, since the electrons delivered to the electrode/solution interface may reduce the oxygen dissolved in the solution. This reaction will be discussed in more detail in Section 2.3. Therefore, in order to determine the photocurrent onset potential the voltammetric measurements were performed in deaerated solutions (to minimize the dark current). The potential of the electrodes was scanned from 0.2 V vs Ag/AgCl towards the negative values, at a low scan rate of 1 mV s^−^^1^ to diminish the capacitance current. The light was chopped at the frequency of about 0.15 Hz.

As visible in Figure 5b,c, the photocurrents reached the dark currents at the potentials −0.28 V vs. Ag/AgCl (i.e., −0.07 V vs. SHE) for g-C_3_N_4_ and 0.02 V vs. Ag/AgCl (i.e., 0.23 V vs. SHE) for C_3_N_3_S_3_. The photocurrent onset potentials were assumed as the Fermi levels (E_F_) of these two materials [35]. Taking into account that the Fermi level of the ambipolar semiconductor is located approximately at the middle of the forbidden band [38] and that the optical band gap of the g-C_3_N_4_ and C_3_N_3_S_3_ determined from the UV-Vis spectra is about 2.9 eV, the estimated values of E_VB_ are of about 1.38 V vs. NHE for g-C_3_N_4_ and 1.68 V vs. NHE for C_3_N_3_S_3_, while the E_CB_ is of about −1.52 V vs. NHE for g-C_3_N_4_ and −1.22 V vs. NHE for C_3_N_3_S_3_, as presented in the band diagram in Figure 5d.

The valence band positions for both polymers were also determined from VB XP spectra presented in Figure 5e. The main absorption onsets are located at 2.1 eV for g-C_3_N_4_ and 2.2 eV for C_3_N_3_S_3_; these values are higher than those obtained from electrochemical experiments. It is worth noting that both XPS spectra also have characteristic tails that cross the energy axes at lower binding energies (at 1.5 eV for g-C_3_N_4_ and 1.73 eV for C_3_N_3_S_3_). Similar features have often been observed in XPS spectra of g-C_3_N_4_, but in general, they were ignored. However, Kang et al. ascribed this tail to the absence of long-range atomic order in the polymer matrix, which results in the dangling bonds, and they determined the valence band position of g-C_3_N_4_ from the tail end [39]. Thus, it is difficult to decide which approach is more appropriate. It should also be taken into account that the state of the semiconductor surface in the electrolyte solution is different than that in a vacuum due to the adsorption of the species at the polymer/solution interface [40]. However, irrespective of the method of determination, the E_CB_ of both polymers is located above the redox potential of H^+^/H_2_, while the E_VB_ is below the oxidation potential of H_2_O to O_2_, which means that g-C_3_N_4_ and C_3_S_3_N_3_ are good candidates for photocatalytic hydrogen and oxygen evolution. The photocatalytic ability of g-C_3_N_4_ in the reaction of water splitting to produce H_2_ has been reported for the first time by Wang et al. [8], but there are several drawbacks, such as limited visible light harvesting efficiency and the fast recombination rate of the photogenerated electron-hole pairs, limiting the hydrogen evolution efficiency on the pristine polymer [41]. A similar problem has been reported for applying g-C_3_N_4_ alone in the photocatalytic degradation of dye pollutants. Therefore, g-C_3_N_4_ is usually combined with other semiconductors, such as TiO_2_, ZnO, WO_3_, Bi_2_WO_6_, and MoS_2_, to reduce the charge recombination (see [2,41,42] and references therein). According to the literature, the g-C_3_N_4_-based heterostructures may also be used for the pyrocatalytic decomposition of dyes [43].

**Figure 6:**
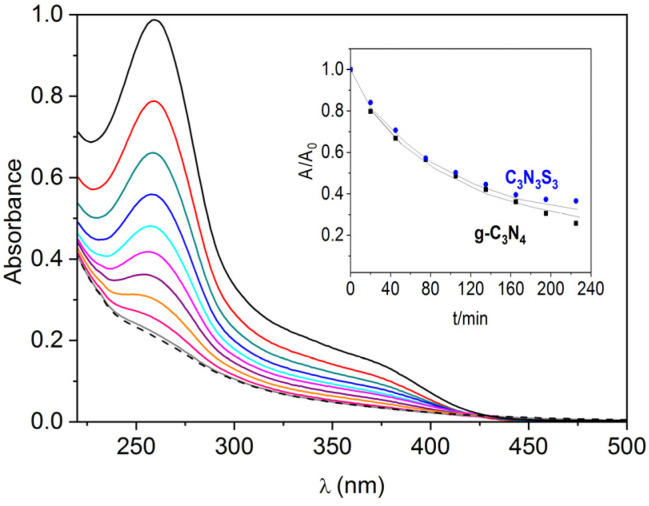
Evolution of UV-Vis spectra of NBT solution under irradiation with a diode 365 nm in the presence of FTO/g-C_3_N_4_ and the change of relative absorbance (A/A_o_) in the peak at 260 nm in a function of time under illumination in the presence of g-C_3_N_4_ and C_3_N_3_S_3_ (inset).

The electrons excited to the conduction band may also be involved in the reduction of oxygen to superoxide anion radical since the redox potential O_2_/O_2_^−•^ (−0.18 V vs. SHE) [44] is located much below the conduction band edges of both polymers (as presented in the band diagram in Figure 5d). The generation of superoxide radicals under the illumination of the g-C_3_N_4_ and C_3_S_3_N_3_ deposited on FTO has been monitored by the changes in the UV-Vis absorption spectra (Figure 6) that occurred due to the reaction of O_2_^−•^ with NBT (as described in Section 3.7). It was found that the rate of transformation of NBT into diformazan was the same for both polymers (inset in Figure 6), which suggests the same ability of both materials in photocatalytic generation of superoxide anion radicals.

### 2.3. Electrochemical Oxygen Reduction and Hydrogen Evolution at the g-C_3_N_4_ and C_3_N_3_S_3_-Modified Electrodes

**Figure 7:**
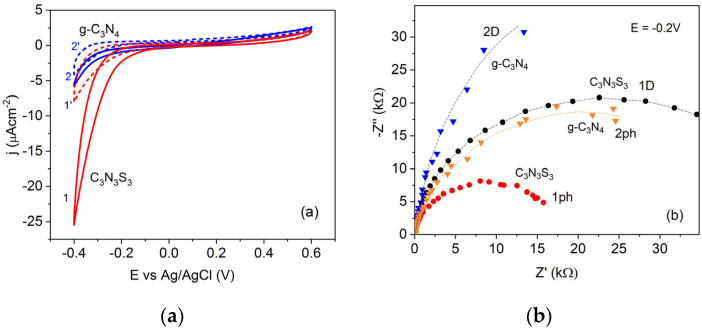
Cyclic voltammograms of C_3_N_3_S_3_ (lines 1) and g-C_3_N_4_ (lines 2) in the solution of 0.1 M Na_2_SO_4_ in the dark, in the presence of oxygen (lines 1,2) and after deaeration (lines 1′ and 2′) (**a**), and EIS curves in the dark (D) and under illumination (ph) recorded at a constant potential of −0.2 V vs. Ag/AgCl in the frequency range 10^5^–0.1 Hz (**b**).

In order to confirm that the cathodic currents recorded in the negative potential range on FTO modified with g-C_3_N_4_ and C_3_N_3_S_3_ (presented in Figure 5a) result from oxygen reduction reaction (ORR), the comparative experiments were performed in the presence of oxygen and in a deaerated solution of 0.1 M Na_2_SO_4_. As visible in Figure 7a, deaeration of the solution led to a significant decrease of the cathodic currents for both polymers (dashed lines), confirming that the oxygen reduction is the main electrode reaction at the semiconductor/solution interface in the potential range from 0 V to −0.4 V vs. Ag/AgCl. It is also worth noting that the process starts at a less negative potential at the C_3_N_3_S_3_ (in the dark and under illumination) than at g-C_3_N_4_ (Figure 5a).

This is also confirmed by the smaller arc radii in the EIS plots obtained for C_3_S_3_N_3_ than that for g-C_3_N_4_ both in the dark and under illumination at the potential −0.2 V vs. Ag/AgCl (Figure 7b). The exact mechanism of oxygen reduction in metals and non-metallic catalysts is still a matter of extensive studies and discussions in the literature [45]. In general, two alternative reaction pathways are considered: a direct four-electron O_2_ reduction or a “serial pathway” with two successive two-electron steps. In acidic solutions, the product of the direct reaction is water, while the two-electron process leads to the formation of H_2_O_2_, which may be further reduced to water in a two-electron reaction. In an alkaline solution, the O_2_ is reduced in a direct four-electron reaction to OH^-^ anions, while in the serial pathway, O_2_ is reduced to peroxide ion $\left({\mathrm{HO}}_{2}^{-}\right)$, which may be followed by either further reduction to OH^-^ or disproportionation to OH^−^ and O_2_ [46,47]. It has also been postulated that the first step of O_2_ reduction on glassy carbon (GC) electrodes in neutral and mild alkaline (pH ≤ 10) solutions is the formation of superoxide anion radical (O_2_^−•^) [48,49].

**Figure 8:**
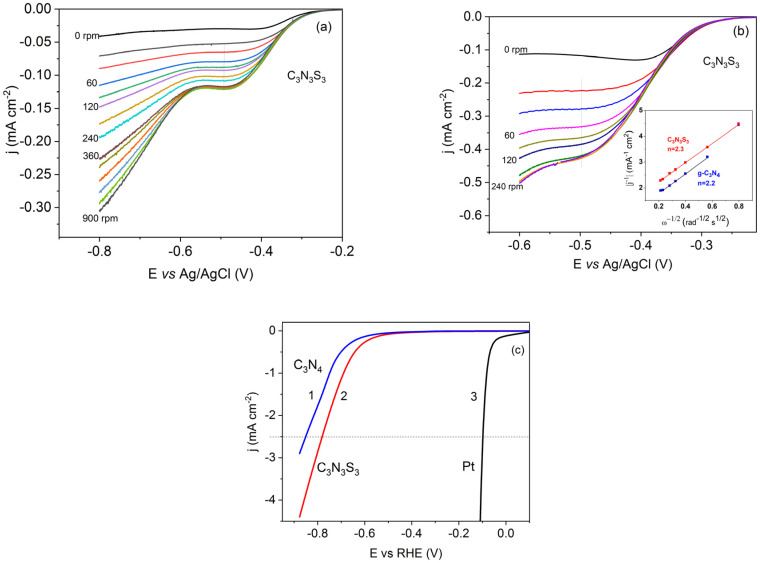
LSVs obtained on GC/C_3_N_3_S_3_ RDE in 0.1 M NaOH at the sweep rate of 2 mVs^−^^1^ and different rotation rates (**a**), and in the same solution after saturation with O_2_ (**b**); inset: Levich–Koutecky plots obtained at C_3_N_3_S_3_ and g-C_3_N_4_-modified electrodes at the potential −0.5 V vs. Ag/AgCl, comparison of the voltammograms for FTO/g-C_3_N_4_ (line 1), FTO/C_3_N_3_S_3_ (line 2) and Pt (line 3) electrodes in 0.5 M H_2_SO_4_ at the scan rate 50 mV/s (**c**).

In order to determine the O_2_ reduction pathway at the g-C_3_N_4_ and C_3_N_3_S_3_ electrodes, linear sweep voltammetric (LSV) experiments using a rotating disc electrode (RDE) were performed. The polymers were deposited on the glassy carbon electrodes by drop cast and polarized in the potential range from 0.1 V to −0.6 V vs. Ag/AgCl at the scan rate of 2 mV s^−^^1^, and different rotation rates in the solution of 0.1 M Na_2_SO_4_. However, no limiting current was observed in the voltammograms recorded in this solution (Figure S4), and therefore, the investigations were carried out in 0.1 M NaOH. For both polymers, the shape of the voltammograms was similar, with a broad wave at about −0.5 V vs. Ag/AgCl, followed by the continuously increasing cathodic current at more positive potentials, as illustrated in Figure 8a. After O_2_ saturation of the solution by oxygen bubbling for 15 min, the limiting current density increased about 4 times (Figure 8b).

The number of electrons (n) involved in the oxygen reduction was determined from the slope of the Levich–Koutecky plot ($j_{lim}^{-1}$ vs. *ω^−^*^1/2^), presented in the inset in Figure 8b, according to the relationship [50]:

(3) $$\frac{1}{j_{lim}}=\frac{1}{j_{k}}+\frac{1}{j_{D}}=\frac{1}{nFkc}+\frac{1}{0.62nFD^{2/3}\nu^{-1/6}c}\cdot \frac{1}{\omega^{\frac{1}{2}}}$$

where *j_k_* and *j_D_* are the current densities controlled by the reaction kinetics and diffusion process, respectively, *F* is the Faraday constant, *c* is a concentration of oxygen in water (1.26·10^−^^6^ mol cm^−^^3^ for O_2_-saturated solution), *D* is the oxygen diffusion coefficient ($D_{O_{2}}=1.96\cdot 10^{-5}$ cm^2^ s^−^^1^), and *ν* is a kinematic viscosity of the solution (0.01 cm^2^ s^−^^1^) (all data from [46]).

The obtained number of electrons, 2.2–2.3 per one O_2_ molecule, suggests the serial ORR pathway for both electrodes (modified with g-C_3_N_4_ and C_3_N_3_S_3_), with the formation of $HO_{2}^{-}$ intermediate in the first two-electron step in the alkaline solution.

Both polymers deposited on the FTO were also used in the preliminary studies of the electrochemical hydrogen evolution in the solution of 0.5 M H_2_SO_4_, deaerated before experiments to avoid the cathodic wave corresponding to the oxygen reduction. As visible in Figure 8c, the hydrogen evolution on g-C_3_N_4_ and C_3_N_3_S_3_ occurs at a much higher overpotential than that at the Pt electrode. On the other hand, the process at C_3_N_3_S_3_ starts a little earlier than at the g-C_3_N_4_ electrode. This may be ascribed to the presence of unsaturated dangling sulfur atoms, which act as the active sites for binding the H^+^ ions. However, since the overpotential of hydrogen evolution at C_3_N_3_S_3_ is large, further modifications, for example, by incorporation of the transition metal ions into the polymer matrix or/and combination with other semiconductors, such as MoS_2_, are needed to improve the catalytic activity of C_3_N_3_S_3_ or C_3_N_3_S_3_-based hybrid system. Thus, the procedures of hybridization of the polymeric and inorganic semiconductor and immobilization of the hybrid system on the surface of FTO to obtain a stable photocatalyst should be developed.

## 3. Materials and Methods

### 3.1. Chemicals

All reagents were of analytical grade and used without further purification. Trithiocyanuric acid (C_3_N_3_S_3_H_3_), Nitro Blue Tetrazolium chloride (NBT), and dimethyl sulfoxide (DMSO) were purchased from Sigma-Aldrich (Darmstadt, Germany). Sodium hydroxide (NaOH, 99.8%), Na_2_SO_4_, H_2_SO_4_, KI, I_2_, urea, and absolute ethanol were purchased from POCh S.A (Gliwice, Poland). The aqueous solutions were prepared using deionized water (DI, 18.2 MΩ cm) (Rephile, Shanghai, China). A conducting FTO (F-doped tin oxide) glass of a resistance 20 Ω square^−1^ was obtained from Dyenamo AB (Stockholm, Sweden).

### 3.2. Chemical Synthesis of C_3_N_3_S_3_

Chemical synthesis of C_3_N_3_S_3_ was carried out according to the procedure described in the literature [16]. Namely, 0.3 g of C_3_N_3_S_3_H_3_ (1.7 mmol) was dissolved in the solution of 0.6 mol/L NaOH under constant stirring for 24 h at room temperature. Then, the temperature of the monomer solution was diminished to 0 °C, and 10 mL of the saturated solution of KI containing 0.65 g (2.56 mmol) of I_2_ was added to maintain the C_3_N_3_S_3_H_3_:I_2_ (oxidant) ratio equal to 1:1.5. Then, the temperature gradually increased to the RT, and the reaction mixture was stirred for 24 h. The obtained yellowish precipitate was washed with deionized water and ethanol. The obtained product was dried at RT and stored in a desiccator. Before application, 6 mg of C_3_N_3_S_3_ was added to 5 mL of DMSO and sonicated for 12 h. The obtained yellowish suspension was stored at the temperature 8 °C. DMSO was chosen as the solvent because of the long-term stability of the prepared suspension.

### 3.3. Chemical Synthesis of g-C_3_N_4_

The urea, used as a precursor of g-C_3_N_4_, was ground in the mortar and placed in a ceramic vessel covered with a lid. The powder was heated in the muffle furnace to a temperature of 500 °C with a ramp rate of 13 °C/min and kept for 1 h. Next, an excess of unreacted urea was washed out with distilled water. Then, 60 mg of obtained pale-yellow g-C_3_N_4_ (60 mg) was suspended in DMSO (50 mL) and sonicated for 10 h in an ultrasonic bath (150 W). The suspension was centrifuged for 10 min at 3000 rpm to remove the non-exfoliated polymer. The obtained white suspension of exfoliated g-C_3_N_4_ in DMSO was stored at the temperature of 8 °C.

### 3.4. Preparation of the Polymer-Modified Electrodes for Electrochemical Measurements

The FTO plates (of the size 2.5 × 1 cm) used as the substrates were washed with acetone by sonication for 15 min. Next, each plate was immersed in the solution of 3M NaOH for 30 s, rinsed with DI water, then immersed for 15 s in the solution of concentrated H_2_SO_4_, and again dipped in DI water. Finally, the FTO plates were dried and used for the deposition of the polymeric semiconductors.

The polymers’ suspensions (600 μL in three portions, 200 μL each) were applied on the FTO substrates by drop casting. After each application, the samples were dried in air. The surface area of FTO covered with the polymers was about 1.5 cm^2^.

A similar procedure was used to modify the glassy carbon (GC) disc electrode with the polymers. The electrode of a surface area of 0.07 cm^2^ was polished by alumina slurry on the felt polishing pad, and then the polymer suspension was applied in three portions of 3 μL each. Roughly, the amount of the polymers applied on GC was about 150 μg cm^−^^2^.

### 3.5. Characterization Methods

The details on the X-ray diffraction and UV-Vis measurements are provided in our recent paper [30] and the Supplementary Materials.

The FTIR spectra were recorded using a Nicolet iS 50 FTIR spectrometer (Thermo Fisher Scientific, Waltham, MA, USA) in the reflection mode in the wavenumber range 4000–350 cm^−^^1^.

The chemical composition and chemical state of the prepared samples were characterized by X-ray photoelectron spectroscopy (XPS). The details on the equipment and data treatment are presented in ref. [51] and in the Supplementary Materials. The measured binding energies for individual elements were corrected in relation to the C1s carbon peak at 284.8 eV.

The binding energy for the valence band (VB) XP spectrum was calibrated using Au of the work function 4.5 eV. This value, typical for a thin polycrystalline gold film [52], practically meets the absolute potential of a standard hydrogen electrode (0 V vs. SHE, i.e., −4.44 ± 0.02 eV vs. vacuum [53]). Therefore, the VB maximum vs. SHE was determined by a linear extrapolation of low binding energy valence band emission edge [54].

### 3.6. Electrochemical Measurements

All electrochemical measurements were performed in a standard three-electrode cell with FTO/g-C_3_N_4_ or FTO/C_3_N_3_S_3_ working electrode, Ag/AgCl (3 M KCl) reference electrode, and Pt plate counter electrode (see details in the Supplementary Materials). The measured potentials were recalculated to the *SHE* scale using the equation:

(4) E(V,vs. SHE)=E(V,vs. Ag/AgCl)+0.21V

The *EIS* measurements were done at the ac voltage of the amplitude of 10 mV, in the frequency range 10^5^–0.1 Hz. In the photoelectrochemical measurements, the working electrode was illuminated with a diode of the wavelength 365 nm.

### 3.7. Detection of Superoxide Radicals O_2_^•−^

**Scheme 1:**
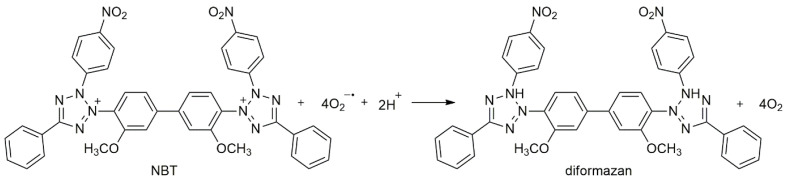
The reaction of Nitro Blue Tetrazolium chloride (NBT) with superoxide radicals [55].

The samples of FTO/g-C_3_N_4_ or FTO/C_3_N_3_S_3_ immersed in an aqueous solution of Nitro Blue Tetrazolium chloride (NBT) of concentration 8·10^−^^3^ g L^−^^1^ was illuminated with a diode (365 nm), under constant stirring. The light intensity in the place of the photocatalyst was 100 mW cm^−^^2^. The electrons photogenerated in the semiconductor reduce oxygen to superoxide radical (O_2_^•−^), which is then involved in the reduction of NBT to diformazan, according to Scheme 1 [55]. In effect, the intensity of the main absorption peak of NBT in the UV-Vis spectrum, observed at the wavelength of 260 nm, decreases.

The reaction rates at different semiconductors were compared by plotting the changes of relative absorbance (A/A_o_) at 260 nm as a function of time, where A_o_ is the initial absorbance of the NBT solution.

## 4. Conclusions

In this work, we have shown that C_3_N_3_S_3_, chemically synthesized from trithiocyanuric acid, reveals very similar electrochemical properties to g-C_3_N_4_. Both polymers exhibit ambipolar behavior, with the Fermi level located in the middle of the band gap. Although the bottom edge of the conduction band in C_3_N_3_S_3_ is located a little below the CB of g-C_3_N_4_, the formation rate of superoxide anion radicals by the photogenerated electrons is the same in both polymers. Both polymers are good electrocatalysts in oxygen reduction, and the reaction starts at lower negative potentials at FTO/C_3_N_3_S_3_ than that at the FTO/g-C_3_N_4_ electrode. The experiments performed using RDE in the solution of 0.1 M NaOH have shown that oxygen reduction occurs via the serial pathway with two successive two-electron steps. The presence of sulfur in the structure of C_3_N_3_S_3_ is probably responsible for the lowering of hydrogen evolution overpotential at this polymer with respect to that at the g-C_3_N_4_-modified FTO electrode due to the presence of additional active sites for binding the H^+^ ions. However, further improvement of C_3_N_3_S_3_ for practical application in HER is necessary.

## Data Availability

Data available on request due to privacy restrictions.

## Supplementary Materials

The following supporting information can be downloaded at: https://www.mdpi.com/article/10.3390/molecules28062469/s1, Figure S1: Survey spectra of C_3_N_3_S_3_ and g-C_3_N_4_; Figure S2: HR-XPS of I 3d and O 1s of C_3_N_3_S_3_; Figure S3: Elemental maps of C_3_N_3_S_3_; Figure S4: LSVs on RDE (C_3_N_3_S_3_) for oxygen reduction; Table S1: XPS data for g-C_3_N_4_; Table S2: XPS data for C_3_N_3_S_3_.

## Sample Availability

Samples of the compounds are available from the authors.
