# Supplementary material for: Comparative Studies of g-C3N4 and C3N3S3 Organic Semiconductors—Synthesis, Properties, and Application in the Catalytic Oxygen Reduction
Source: Molecules. 2023 Mar 8;28(6):2469. doi: 10.3390/molecules28062469 (PMC10058859; doi:10.3390/molecules28062469)
Supplement: Supplementary file 1 [file molecules-28-02469-s001.zip › molecules-2257435-supplementary.pdf]

Supplementary materials

Comparative Studies of g-C_3N_4 and $C_3N_3S_3$ Organic Semiconductors—Synthesis, Properties, and Application in the Catalytic Oxygen Reduction

Ewelina Wierzyńska [1], Marcin Pisarek [2], Tomasz Łęcki [1] and Magdalena Skompska [1,*]

[1] Faculty of Chemistry, University of Warsaw, Pasteura 1, 02-093 Warsaw, Poland
[2] Institute of Physical Chemistry, Polish Academy of Sciences, Kasprzaka 44/52, 01-224 Warsaw, Poland
* Correspondence: mskomps@chem.uw.edu.pl

Characterization methods

The crystalline structure of the samples was determined by X-ray diffraction using a broad angle XRD diffractometer (Bruker D8 Discover, Germany). The spectra were collected in the 2θ range from 10° to 60°.

Diffuse reflectance spectra (DRS) of the samples were recorded using a UV-Vis spectrometer (Shimadzu UV-3600) equipped with an integrating sphere. The spectrophotometer Lambda 12 (Perkin Elmer) working in a transmission mode was used to monitor the changes in the concentration of NBT solutions used for detection of superoxide anion radicals ($O_2^{\bullet -}$).

The chemical composition and chemical state of the prepared samples were examined with XPS, by means of PHI 5000 VersaProbe (ULVAC-PHI) spectrometer. The XPS spectra were excited using $Al_{K\alpha}$ (hν = 1486.6 eV, 25 W) monochromatic radiation as a source, at a resolution of binding energy 0.1 eV. The survey and high-resolution (HR) spectra were collected at a constant pass energies of 117.4 and 23.5 eV, respectively. Avantage Surface Chemical Analysis software – Thermo Fisher Scientific (ver. 5.9911) was used for the data processing. The background was corrected using the Smart model to obtain the XPS signal intensity. An asymmetric Gaussian/Lorentzian function at a constant ratio G/L = 0.35 was used for the deconvolution procedure.

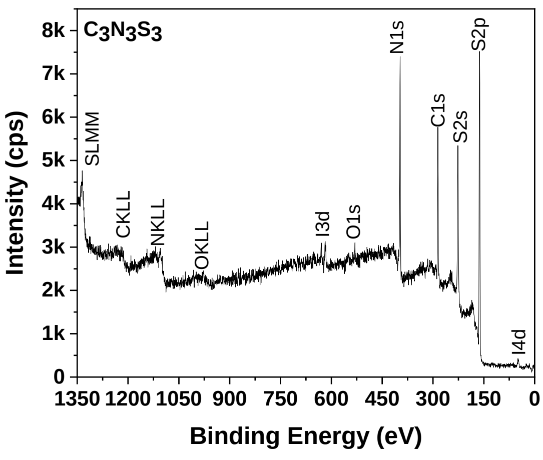

(a)

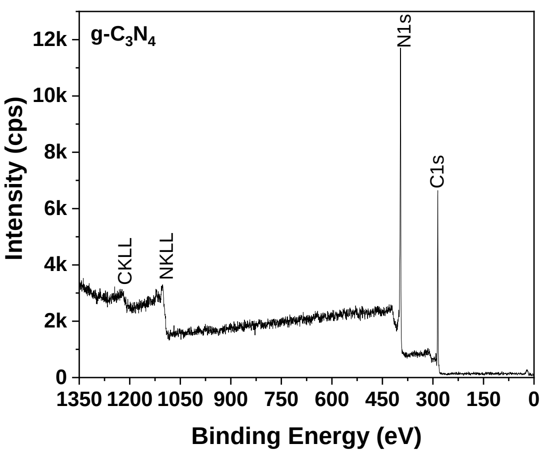

(b)

Figure S1. Survey spectra of $C_3N_3S_3$ (**a**) and g-C_3N_4 (**b**).

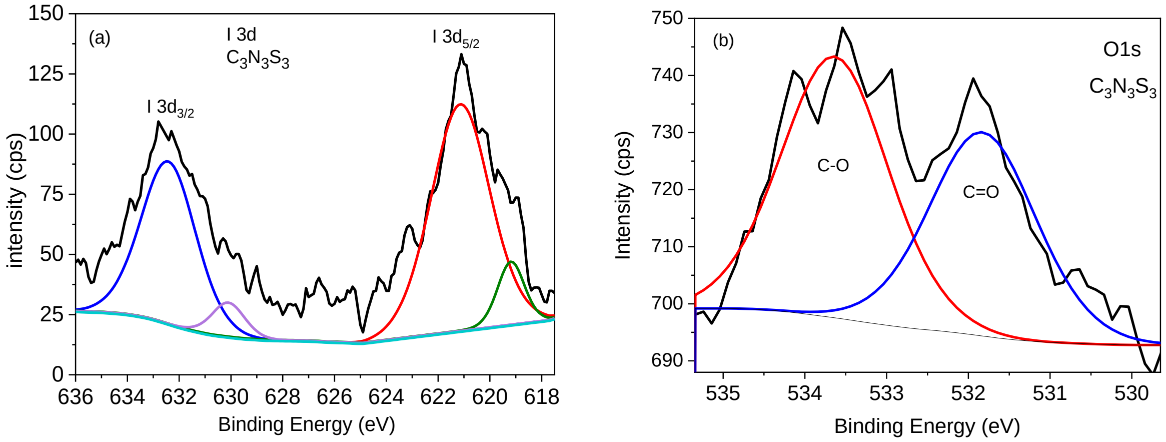

Figure S2. High resolution XPS spectra of I 3d (**a**) and O 1s (**b**) of $C_3N_3S_3$

Table S1. The XPS data for g-C_3N_4.

Name	Peak BE / eV	Atomic %	chemical bonds
C1s	288.1	36.8	N-C=N
C1s	289.0	3.0	C-N-C
C1s	284.8	0.8	C-C
C1s	286.3	0.7	C-N
C1s	293.5	2.8	shake up satellites
N1s	398.6	38.5	N-C=N
N1s	399.9	8.2	N-$(C)_3$
N1s	401.0	6.3	C-N-H
N1s	404.3	2.0	shake up satellites
N1s	406.2	0.9	shake up satellites

100.0

Table S2 The XPS data for $C_3N_3S_3$

Name	Peak BE / eV	Atomic %	chemical bonds
C1s	287.9	27.8	N-C=N / C-S
C1s	289.0	2.8	C-N-C and O=C-OH
C1s	284.8	1.5	C-C
C1s	293.0	0.9	shake up satellites
C1s	285.9	0.7	C-N
N1s	399.6	24.5	N-C=N
N1s	400.7	2.1	C-N-H
N1s	404.2	1.2	shake up satellites
N1s	407.5	0.5	shake up satellites
O1s	533.6	0.5	C-O
O1s	531.8	0.4	C=O
S2p3/2	164.7	24.6	S-S (disulfide) / C-S
S2p1/2	165.9	12.4	S-S (disulfide) / C-S
I3d5/2	621.2	0.1	I_2
I3d3/2	632.5	0.1	I_2

100.0

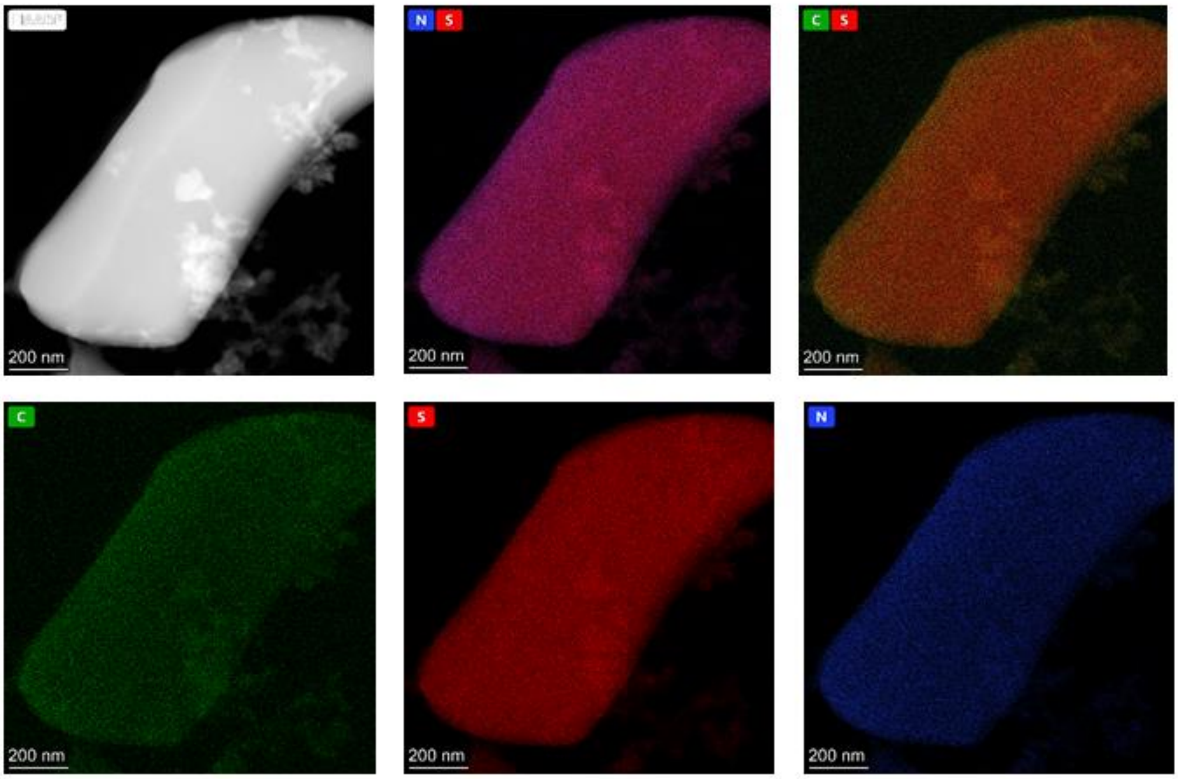

Figure S3. EDS elemental maps of $C_3N_3S_3$

Electrochemical measurements

All electrochemical measurements were performed in a standard three-electrode cell with FTO/g-C_3N_4 or FTO/$C_3N_3S_3$ working electrode, Ag/AgCl (3 M KCl) reference electrode, and Pt plate counter electrode. The potential of the reference electrode determined from the Nernst equation is 0.21 V vs. SHE (standard hydrogen electrode).

Cyclic voltammetry (CV), linear sweep voltammetry (LSV) and electrochemical impedance (EIS) experiments were done with the use of Autolab PGSTAT 20 (Metrohm, Netherlands) with FRA module.

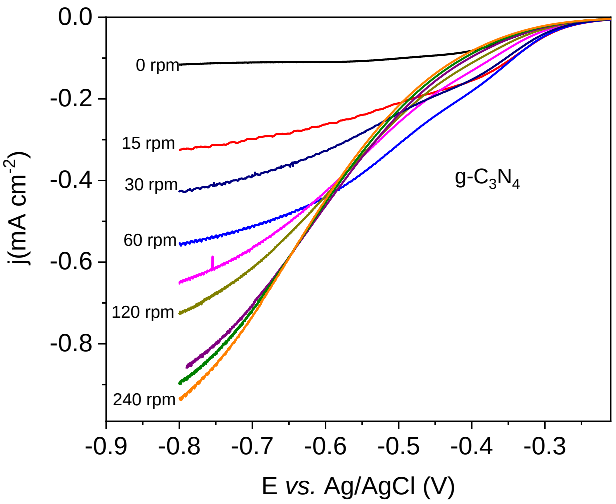

Figure S4. LSVs obtained on GC/$C_3N_3S_3$ RDE in the solution of 0.1 M Na_2SO_4 saturated with O_2, at the sweep rate 2 mVs^{-1} and different rotation rates.
